# Telemedicine in cancer care: lessons from COVID-19 and solutions for Europe

**DOI:** 10.1093/eurpub/ckae206

**Published:** 2025-01-03

**Authors:** Anita Gottlob, Tugce Schmitt, Morten Sønderskov Frydensberg, Magdalena Rosińska, Victoria Leclercq, Katharina Habimana

**Affiliations:** Gesundheit Österreich GmbH, National Institute of Public Health, Vienna, Austria; Cancer Centre, Sciensano, National Public Health Institute, Brussels, Belgium; Department of Digital Innovation, Health Innovation Centre of Southern Denmark, Odense, Denmark; Department of Computational Oncology, Maria Sklodowska-Curie National Research Institute of Oncology, Warsaw, Poland; Cancer Centre, Sciensano, National Public Health Institute, Brussels, Belgium; Gesundheit Österreich GmbH, National Institute of Public Health, Vienna, Austria

## Abstract

The COVID-19 pandemic challenged healthcare delivery, especially cancer care. Telemedicine emerged as an important tool to reduce disease transmission risks, maintain continuity of care, and improve accessibility. This study explores temporary measures during the pandemic as well as challenges and facilitators for integrating telemedicine into the European healthcare landscape in five case countries, focusing on cancer care. Expert interviews were conducted in five EU countries with diverse health systems: Austria, Belgium, Denmark, Italy, and Poland. A thematic analysis was performed. Themes were further explored related to regulatory changes during COVID-19 as well as barriers and facilitators to telemedicine implementation. COVID-19 accelerated telemedicine uptake and processes (i.e. regulations, reimbursement) in all case countries. Acceptance of telemedicine increased among healthcare professionals and patients. Post-pandemic telemedicine use and acceptance declined to pre-pandemic levels in some countries and was attributed to several factors including preferences for in-person visits. Overall, persistent barriers were identified by all country experts including lack of standardized policies, data privacy concerns, technological infrastructure issues, and digital literacy gaps. Telemedicine was validated by all country experts as an important tool to enhance cancer care access and efficiency and to help maintaining continuity of cancer care during crises. Our findings highlight some overlapping barriers and suggest solutions to overcome these barriers across the selected countries. Recommendations for policymakers are listed, emphasizing the importance of telemedicine services in improving healthcare access, efficiency, and resilience. Future research should incorporate diverse population studies, patient perspectives, cost-effectiveness, and policy impacts.

## Additional content

Additional content An author video to accompany this article is available at: https://oup.cloud.panopto.eu/Panopto/Pages/Viewer.aspx?id=b38255ea-2d8a-4611-a3f5-b23c008820c3.

## Introduction 

The COVID-19 pandemic posed unprecedented challenges across healthcare systems worldwide, leading to a reduction in in-person services and exacerbating delays in critical medical treatments, including cancer care [1–3]. This impact varied across Europe, influenced by differences in healthcare infrastructures, levels of digital maturity, and regulatory frameworks. Therefore, many countries implemented short-term deregulation of telemedicine (TM), sometimes leading to long-term changes.

TM, the remote delivery of healthcare services via information and communication technology (ICT) [4], played a vital role in mitigating the spread of COVID-19 while ensuring continuity of care where possible. During the crisis, the advantages of TM became evident, including reducing disease transmission risk, optimizing resource allocation, and expanding healthcare access through video-based consultations [5, 6]. Additionally, TM has increased infrastructure efficiency [7]. These benefits are particularly relevant for immunocompromised cancer patients, as research indicates TM’s cost-effectiveness, high-quality care, and improved accessibility compared to in-person care [8]. However, TM’s effectiveness varies by cancer stage and type, sometimes requiring a hybrid or in-person approach [9, 10].

TM offers significant benefits for patients in rural or remote areas, those with socio-economic constraints, or limited mobility [11, 12]. For instance, TM helps low-income patients overcome barriers such as transportation costs or childcare needs during consultations [11, 13]. However, the efficacy of eHealth tools like TM is contingent on individuals’ access to and proficiency with these technologies, creating a ‘secondary digital divide’ in health services [14]. Low digital literacy among cancer patients can further disadvantage those already facing socio-economic challenges [7, 14].

The structure of the health system, national income, healthcare budgets, geographical aspects, ethical considerations, and the complexity of regulatory procedures all add layers of complexity to the implementation of ICT and TM [15–18]. Ethical and safety considerations are critical factors that decision-makers must address when adopting TM services [15, 17, 18]. Despite the surge in TM adoption during the pandemic, its long-term integration remains inconsistent, and its potential underexplored [12].

This paper aims to explore the following research question: How have the disruptions caused by COVID-19 influenced ongoing TM policies, and what barriers remain to be overcome to facilitate broader adoption of TM across European countries?

To address this question, we investigate the challenges and strategies for TM implementation in cancer care across Europe, using a qualitative approach through expert interviews in five EU countries with diverse eHealth implementations and healthcare system structures: Austria, Belgium, Denmark, Italy, and Poland.

## Methods

We employed a qualitative research design using expert interviews to gather in-depth insights into the impact of COVID-19 and enduring barriers, as well as facilitators of TM implementation with a focus on cancer care across five EU countries: Austria, Belgium, Denmark, Italy, and Poland. Countries were intentionally selected to cover different European regions and health system setups. The case countries reflect diverse approaches to eHealth, including TM in general and during the COVID-19 pandemic. An overview of comparative indicators for eHealth related to the case countries is available in the Supplementary Materials. We would also like to note that an enterprise version of Microsoft copilot was used to check the accuracy of grammar and spelling of some parts of the paper.

### Data collection

Semi-structured expert interviews were conducted with 10 key informants in total, of which two were in Austria, two in Belgium, three in Denmark, and two in Italy. We aimed to collect information from at least one leading expert per country ideally working at the governance level. Interview questions are available in the Supplementary Materials (Box S1). Inclusion criteria for experts were based on their professional experience in the field of eHealth, including publication of strategic and operational documents; involvement in drafting of or consulting national regulatory frameworks related to TM; drafting legal acts in eHealth; contributions to national strategies, with specific knowledge on TM strategies and implementation.

Where possible, the experts provided evidence in support of their statements, which is cited throughout the results and discussion of the manuscript.

### Ethical considerations

The Interview process followed ethical guidelines for research involving human participants, ensuring confidentiality and voluntary participation. Informed consent was received from all participants prior to the interview, ensuring that they were aware of the study’s purpose and their involvement. To protect the anonymity of the experts, we removed all personal identifiers from transcripts and analyses. Data is presented in aggregate form and no personal data about the interviewed experts is shared.

### Thematic analysis

Following the main steps of thematic analysis loosely based on Clarke and Braun [19]. The content from transcribed interviews and written answers was first read and vertically coded manually—both deductively and inductively—throughout the dataset to identify commonly appearing topics. The codes were reread and combined into themes subsequently validated by the co-authors of the study. This latter stage was aided by an enterprise version Microsoft-Copilot, ensuring confidentiality and stringent data protection standards. Microsoft Copilot is based on the GPT-4 architecture and is well suited for thematic analysis due to its advanced natural language processing (NLP) techniques, ensuring consistent application of coding and theme identification criteria, reducing the risk of human error and bias. he final themes, which closely resembled the interview question topics (see Box S1 in Supplementary Materials): (1) pre-COVID-19 situation, (2) COVID-19 acceleration, (3) perceived benefits from TM, (4) barriers and challenges, and (5) facilitating factors and solutions.

### Content analysis

We then conducted a content analysis to identify the most frequent codes related to the themes of ‘barriers and challenges’ and ‘facilitating factors and solutions’. Here, Microsoft Copilot was used to count the occurrence of each code related to the two themes (priorly identified manually). This provided a synthesized and structured overview of the data, focusing on the barriers and challenges in TM implementation. The suggested solutions were summarized into general recommendations. Codes were validated by the co-authors of the study to ensure validity and reliability.

## Results

The summarized themes across all case countries (Table 1) include information on; TM before the COVID-19 pandemic, changes of TM perception, use, regulations that occurred due to COVID-19, perceived benefits of TM during COVID-19 and in general, barriers and challenges to TM use and implementation and perceived facilitators in the national context.

**Table 1.** Overview of results along the five themes

Theme	Austria	Belgium	Denmark	Italy	Poland
Pre-COVID-19	TM permitted but not fully integrated. No specific policies for TM use in cancer care. TM broadly regulated in the agreement made under article 15 in the Austrian federal Constitution ‘15-A Vereinbarung’ (renewed in 2023) [20] 1450 hotline as an example of a regional TM service in Austria (Vienna) [21]	No specific policies for TM use in cancer care. Lack of structured reimbursement for TM.	National focus on strengthening digitization of the healthcare sector and deployment of TM since 2012. Efforts since 2015 for a national joint TM platform [24] Regulatory frameworks for the use of TM not pathology specific e.g., on cancer care. TM use part of the healthcare system with some reimbursement. TM infrastructure aimed at economies of scale.	General regulatory framework since 2014 [25] No specific TM regulations in oncology. TM not widely and unevenly implemented particularly in cancer care. Longstanding healthcare personnel shortage.	General definition of TM in the national regulations (Act on Medical Activity) [29] No specific guidelines of TM and cancer care. Teleconsultations are complementary not a substitutive element of the Polish healthcare system. Teleconsultation legally approved as one of the guaranteed services of primary health care (public health insurance services). Teleconsultations used for e-prescriptions. Teleconsultations possible also by nurses for referrals for higher level of care. No specific TM provisions for cancer care.
COVID-19 Acceleration	Both short- and long-term changes in legislation triggered by COVID-19. Financial and regulatory state-level changes were made to support and potentially expand TM services. Increased integration and acceptance of TM. Adaptations to the hotline 1450 for telehealth consultations on COVID-19 symptoms and sick certifications. Increased mention of TM in healthcare agreements specifically in the '15a-Vereinbarung' [20]	Both short- and long-term changes in legislation triggered by COVID-19. Temporary and later structural reimbursement policies for teleconsultations for general practitioners and specialists. Rapid adoption of TM to reduce hospital pressure and provide patients with quick access to healthcare services during the pandemic. Royal decrees were implemented to adjust TM practices and incorporate them permanently into the healthcare system [23]	Intensification of existing TM projects. Increased use during lockdown periods. Continued consultations during lockdowns via tele-interpreting. Expansion of TM reimbursement schemes which are planned to be maintained.	Both short- and long-term changes in legislation triggered by COVID-19, such as ministerial guidelines in 2021 for telerehabilitation services covering all remote services for people with disabilities and/or disorders [26] New regulations supporting tele-rehabilitation and structured TM implementation. Interim guidelines facilitated immediate TM use. Efforts to establish a national TM platform by 2025 [27] Significant financial investments in the framework of the National Recovery and Resilience Plan (NRRP) aim to standardise TM use [28]	Both short- and long-term changes in legislation triggered by COVID-19. Enhanced financial and regulatory support such as reimbursement structures established at state level. Temporary regulations facilitating remote healthcare services. *Ad hoc* integration of TM in oncology.
Perceived Benefits	Interest in teletherapy grew among therapists. Potential for expanded TM services.	Maintained healthcare accessibility during the pandemic. Enhanced accessibility to services for patients. Potential customization of reimbursements for specific pathologies.	TM as a complement to traditional services. Potential for patient empowerment in health management.	TM is seen as potentially beneficial across all stages of cancer care though practical application remains limited.	Management of chemotherapy and drug programs remotely: TM was integrated *ad hoc* into oncological care supporting remote management of treatments.
Barriers	Digital competency gaps. Legislative clarity needed. Data protection and standardization concerns.	Scepticism about TM’s effectiveness in practitioners and patients. Interoperability and data security concerns. Access to technology. Varying degrees of digital literacy.	Economic challenges in local funding of TM platforms. Difficulty in tracking benefits and savings across sectors. Lack of digital health literacy among patients and HCPs.	Technological knowledge and infrastructure limitations. Uneven digital literacy among providers and patients. Lack of a unified technological infrastructure.	Digital health literacy and technological limitations. Public awareness and potential dehumanization concerns. Need for legislative adjustments and training.
Facilitators	Adoption of the European Health Data Space (EHDS)—allowing for interoperability and standardization of TM solutions [22]	Interoperability and standardization of TM solutions. Current efforts to implement structural reimbursements for teleconsultation services. Education and training on TM use.	Sustained political will and strategy are crucial for overcoming economic barriers and supporting long-term TM integration.	Centralized national TM platform facilitating interoperability and monitoring [27] Financial investments in the framework of NRRP [28]	TM Roundtable with diverse stakeholders for regulatory development. National digital transformation of healthcare as a priority. Public funding for TM and a national focus on digital healthcare transformation.

### COVID-19 impact

The interviews revealed that the COVID-19 pandemic impacted the use of TM across all countries on several levels. All country experts, except those from Denmark, reported specific temporary changes in regulatory frameworks and reimbursements leading to long-term changes or current developments in this direction. In contrast, Denmark, which had a national focus on TM before 2012, already had regulatory frameworks for the use of TM, reimbursement agreements for TM services, and numerous projects and initiatives underway before the pandemic (although few specific to cancer). Interviewees perceive that the ‘momentum of change’—as expressed by one expert—was challenging to maintain in comparison to the time during the pandemic. Nevertheless, Danish experts mentioned that there is still a significant demand for digital services from patients.

Similarly, Polish experts noted a decline in TM usage and interest among healthcare professionals (HCPs) and patient’s post-pandemic. Please note that no specific reference was given for this statement, which we consider to be an expert opinion. In Poland, regulations were introduced within the legal framework for pandemic control and were therefore temporary. The Interviewees referenced a report showing that during the pandemic, 80% of consultations in Poland were teleconsultations, which declined to 31% in primary health care and 13% in specialist health care by 2022 [30]. Nonetheless, the pandemic spurred the digital transformation of healthcare in Poland, which is considered a key enabler for TM. A facilitator in the experts’ opinion was cooperation across stakeholders via the establishment of a roundtable involving public administration, medical professionals, NGOs, and IT system providers, working on developing principles, guidelines, best practices, and framework documents to shape TM regulations.

Austrian experts highlighted the regional 1450 hotline (Vienna), used for general teleconsultations before the pandemic and quickly adapted for teleconsultations to diagnose symptoms in suspected COVID-19 cases and provide further consultation [21]. This service later reverted to its original use. Interviewees also referenced a 2020 survey showing that the pandemic increased TM acceptance among medical professionals: 61% saw a large potential for TM during crises, 57% supported its use, 34% were sceptical, and 8% rejected TM use [31]. While comprehensive data on TM use in Austria is lacking, the Austrian National Public Health Institute is tasked with providing an overview of TM service developments. The newly adopted European Health Data Space (EHDS) regulatory framework is expected to improve the monitoring of actual data and facilitate TM service implementation by addressing interoperability and other challenges [22].

Belgian experts noted that TM was introduced in Belgium to alleviate hospital pressure during the pandemic and maintain healthcare accessibility. They emphasized that TM was especially useful in this respect and suggested that it could enhance access to services for patients. Ongoing discussions aim to customize TM service reimbursements to meet the specific needs of various pathologies, such as telemonitoring patient data for heart disease. However, political decisions regarding TM currently adopt a universal approach, lacking specific policies dedicated to cancer care.

Italian experts note that the pandemic served as a critical catalyst for extending the reach and policy support for TM services, including tele-rehabilitation and a more structured approach to TM deployment noted in several new regulations established during the pandemic. Specifically, for oncology, TM services are guided by national regulations encompassing a wide range of services from prevention to palliative care. The National Recovery and Resilience Plan (NRRP), part of the Next Generation EU program allocates €2.5 billion for investments in healthcare system digitalization, including €1 billion specifically for TM implementation [28]. The NRRP reforms position TM as a central component in the new model for localized healthcare organization. This is seen as a facilitator, along with the development of a centralized national TM platform by the Italian Ministry of Health, which aims to bridge territorial disparities (expected in 2025) [27]. It will validate/monitor TM solutions implemented by regions and autonomous provinces through their existing systems, provided they are interoperable.

### Barriers and proposed solutions

Country-specific barriers and facilitators in the national context are listed in Table 1. In addition, Table 2 summarizes the overlapping barriers (pertaining to the post-pandemic period) across the case countries. The list below shows the barriers that were most to least frequently mentioned across the responses from the five case countries. These were clustered into five categories:

**Table 2.** Summary of common barrier categories and suggested solutions/recommendations

	Mentioned categories of barriers	Suggested solutions
Austria	#Digital Skills, #Legislation, #Accessibility	Clarify regulatory framework, invest in infrastructure, address data protection, promote education, encourage collaboration.
Belgium	#Preference, #Data Challenges, #Digital Skills, #Accessibility	No explicit recommendations were given
Denmark	#Legislation, #Digital Skills, #Preference	Invest in user-centred solutions, IT support, patient empowerment, improve digital competencies, embrace holistic value creation, political support
Italy	#Accessibility, #Data Challenges, #Digital Skills	Investing in health literacy skills, investing in digitalization
Poland	#Digital Skills, #Accessibility, #Preference, #Legislation	Investing in digital transformation, digital literacy and skills training for HCPs and patients, development of information campaigns

#### Digital skills

This barrier encompasses the lack of digital competencies among HCPs and patients, particularly older individuals. Mentioned by all countries, indicating a widespread need for improving digital competencies among both HCPs and patients.

#### Accessibility

Noted by all countries, highlighting issues with the availability and centralization of TM services and relevant infrastructure. Patients and Healthcare providers often face difficulties accessing these services due to a lack of streamlined platforms and scalable business models in the public sector, as well as issues such as ICT.

#### Legislation

Cited by Austria, Denmark, and Poland, reflecting concerns about the legal framework surrounding TM, including issues of liability, data protection, and reimbursement issues.

#### Preference for in-person visits

Mentioned by Belgium, Denmark, and Poland, showing hesitancy among HCPs and patients towards TM.

#### Data challenges

Brought up by Belgium and Italy, pointing to issues with data security, interoperability, management of telemonitoring data.

Italian experts note that Italy experiences one of the most substantial digital divides in the European Union: The latest DESI (Digital Economy and Society Index) report in 2022 ranks Italy among the lowest for digital skills. Moreover, elderly and chronic patients, who would benefit most from remote care, often face greater difficulties using technological devices. The progress of TM has therefore been significantly hindered by patients’ challenges in using computers.

In another example, Danish interviewees noted that while political priority has been given to TM, the advantages of such investments can be challenging to discern in the short term without sustained political will and strategy. They stress that the benefits of TM are often overlooked in economic/financing decisions both on a national and local level:

At a local level, integrating TM can be complex. It is often clearer where the costs lie than where savings occur, particularly when coordination across different sectors is needed:

Overall, most countries emphasize the need for investment in infrastructure, education and training, data protection and security, interoperability and standardization, and political support with a long-term strategy. Belgian experts did not give explicit potential solutions to the barriers they mentioned; however, education and training were mentioned as important aspects of TM implementation.

## Discussion

In this work, we aimed not only to describe the temporary measures instituted during the pandemic in case countries but also to understand the enduring barriers and possible facilitators to a comprehensive implementation of TM into the European healthcare landscape, particularly in cancer care.

It must be noted that this study has certain limitations; firstly, the findings are based on expert opinions, which may introduce subjectivity and selection bias, limiting the generalizability of the results. Secondly, the interviews were conducted in a small number of countries, in accordance with the available resources and practicability of performing the research within the eCAN Joint Action project, therefore limiting a diversity of perspectives, for example by smaller or eastern European countries. Nevertheless, the qualitative insights provided are valuable as they complement existing literature and research by highlighting the barriers and facilitators identified by experienced stakeholders in the field, thereby offering a deeper understanding of the practical challenges and opportunities in sustainably integrating TM into cancer care.

Our results show that experts in the field consider COVID-19 to have had considerable impact on (1) acceleration in processes around TM regulation, guidelines, and funding as well as thoughts about reimbursement of TM solutions; (2) increased acceptance, awareness, and use of TM solutions by health professionals and patients alike; and (3) helping to maintain continuity of care in times of crisis for specific patient groups such as already immunocompromised cancer patients.

The interviewees stressed the benefits of TM during COVID in their countries, notably in maintaining continuity of care where possible and showed a unified favourable attitude towards improving the implementation of TM service in general and in cancer care. The findings are congruent with recent OECD data, where country experts agree that TM services may help to enhance equity, efficiency, access, cost-effectiveness, and quality of healthcare [32].

However, considerable overarching barriers still exist.

Based on the results and literature in this field, we suggest that these barriers are often inter-related/may influence each other. For example, on a governance level, regulatory barriers were mentioned, including the absence of standardized TM policies across countries, creating challenges due to divergent regulations concerning patient data protection and privacy. Data privacy itself also relates to TM acceptance, as people may be hesitant to use TM services if they have concerns about data being shared or stored in insufficiently secure environments. Recent literature underlines that policy constraints such as inconsistent regulations regarding patients’ rights, medical practice conditions, and data management may clash with policy enablers such as prior commitments that create trust, security, and confidentiality in TM [17]. Furthermore, technological barriers related to infrastructure, such as the lack of consistent and reliable ICT infrastructure or internet connectivity can hinder an effective implementation of TM services. Similarly, unequal access to the Internet, what is termed the ‘digital divide’, has been shown to influence the utilization of eHealth [33]. This may affect isolated, for example, communities in remote areas, which can both necessitate and complicate the implementation of TM services if the internet connection is not optimal in those regions [34].

Studies determined that beyond this gap in access, knowledge related to the use of the Internet also has an impact on the use of eHealth [14, 34]. This may affect especially older patients [35] and chronic disease patients [36], who would benefit most from remote care. Both groups often face greater difficulties using technological devices; For older adults, challenges can include reduced cognitive abilities, lack of familiarity with technology, and physical limitations as well as anxiety or resistance towards using new technologies [37]. For patients with chronic diseases different, more procedural challenges may arise for example due to the burden of managing multiple health conditions [36]. Low digital literacy, along with structural barriers, were noted by all case countries as major challenges to TM implementation. Conversely, TM has also been shown to have the potential to serve as a bridge for equitable access by addressing barriers for various demographic groups, particularly socially disadvantaged populations [11]. For example by decreasing costs for patients and caregivers significantly, particularly in travel and accommodation, and for patients with limited means in general [5, 9, 36, 37]. Therefore, policies around TM implementation should consider that the adoption of eHealth technologies, has both the potential to reduce social health inequalities, and at the same time also to increase them for population groups who do not have access or digital skills to use these tools [14]. This highlights the need for a holistic, user-centred, and strategic approach to address both primary and secondary digital divide via (1) interventions to increase access and (2) developing digital skills via targeted comprehensive training and education for those who need it.

In some countries, a decline in post-pandemic TM use was attributed to potential preference for in-person consultations among both patients and HCPs. The interviewees also mentioned that hesitancy towards TM use can arise due to various cultural barriers. Studies show that in-person visits may be better for certain cancer patients, particularly children and those with complex or metastatic cancers, who require more in-person care. These factors should be further examined and limiting factors of TM use for cancer patients should be considered when training HCPs and informing patients on TM advantages and disadvantages. For example, literature in this field shows that concerns about TM’s effectiveness and quality in stages such as post-chemotherapy and long-term survivorship highlight the need for nuanced TM applications in cancer care [5, 38, 39].

While COVID-19 expanded the necessity and awareness of eHealth services, maintaining momentum depends on stakeholder cooperation and involvement. Some interviewees noted that key stakeholders, such as HCPs sometimes remain hesitant to apply for TM funding, on a local level, due to perceived short-term expenses. The inclusion of key stakeholder, such as physicians, patient associations, pharmacies, nurses, and private health industry actors, can either facilitate or hinder TM adoption, depending on the organization of the health system [16, 17, 19]. In Poland, the TM Roundtable exemplifies successful stakeholder involvement in TM implementation. Ultimately, despite initial disparities in their pre-COVID-19 setups, all countries successfully expanded the use of TM services since the COVID-19 pandemic.

In conclusion, our results suggest that the strategic use of TM for specific patient groups can help alleviate pressure on the national cancer care system in times of crises. It has emerged as a transformative tool for cancer care delivery across European countries with the potential to address critical challenges in different areas. It may become an essential, highly reliable method of physician consultation during health crises such as the COVID-19 pandemic [15, 16]. Nonetheless, ensuring equal access to such innovative services is crucial. To enable this, barriers related to infrastructure, accessibility, and education on digital skills need to be addressed. Moreover, a sustainable, ethical, and holistic approach to TM implementation is needed to create the conditions for equal access to users/patients.

Based on the barriers identified across the five case countries and in literature, we suggest the following recommendations to policymakers for the development, implementation, and use of TM services on a national level:

1. Establish a clear legal framework on development, data aspects, certification, and ways to integrate TM into clinical care, through interdisciplinary collaboration with relevant stakeholders, addressing data protection and security issues.
2. Improve education on digital skills of HCPs including training on data security and competency to determine in which cases TM can guarantee the same quality of care as in-person visits.
3. Improve patient access to technology and digital skills with particular focus on cancer patient groups with low digital health literacy skills.
4. Invest in infrastructure and develop Information Technology (IT) infrastructure to create an environment conducive to TM services, especially in healthcare-deprived and rural areas.
5. Improve interoperability and standardization via creation of central TM data infrastructures and platforms to reduce fragmentation while ensuring data protection.
6. Consider the importance of political support and strategic policies emphasizing the need for sustained political will and the establishment of a holistic understanding of the value of TM in the health system.
7. Provide sustainable financing and long-term investments in TM such as funding for research as well as sustainable reimbursement mechanisms.

Future research should prioritize examining which target populations need digital skills enhancement and identifying effective methods to increase TM accessibility and skills among these groups. Additionally, comprehensive cost-benefit analyses are needed to evaluate the optimal use of TM in cancer care, including the most effective allocation of resources and funding. Investigating these areas will provide critical insights into the sustainable implementation of TM and its potential to improve patient outcomes and healthcare efficiency.

## Supplementary Material

ckae206_Supplementary_Data

## Data Availability

The data (interview transcripts) underlying this article cannot be shared publicly for the privacy of the individuals who participated in the expert interviews. The data will be shared on reasonable request to the corresponding author. Key points Our findings indicate that TM can alleviate pressure on national cancer care system during crises and help to maintain continuity of care for specific patient groups, such as immunocompromised cancer patients. This research suggests that improving digital competencies among HCPs and patients is crucial to provide equal access to TM services. Policymakers should create conditions for equal access by developing a supportive regulatory framework and improving acceptance among healthcare providers and patients. Investing in technological infrastructure, training, education, and fostering stakeholder collaboration is essential to overcome barriers to TM implementation.
