# Supplementary material for: Telemedicine in cancer care: lessons from COVID-19 and solutions for Europe
Source: Eur J Public Health. 2025 Jan 3;35(1):35–41. doi: 10.1093/eurpub/ckae206 (PMC11832154; doi:10.1093/eurpub/ckae206)
Supplement: ckae206_Supplementary_Data [file ckae206_supplementary_data.docx]

**Supplementary Material to the Manuscript: Telemedicine in Cancer Care: Lessons from COVID-19 and Solutions for Europe**

**Box S1. Interview themes and questions**

| **Interview Questions** |
| --- |
| ***Q1:*** *What is the current situation of telemedicine services for cancer patients in your country?*  ***Q2:*** *Was policy for telemedicine/teleconsultation changed during COVID? What was changed and was it included in any documents (laws, directives, guidelines etc.)?*  **Q3:** *Which stakeholders were involved in/contributed to decision making in telemedicine regulations (did it include cancer care/oncology experts)?*  ***Q4:*** *What barriers exist in terms of increasing telemedicine services in your country and how could they be improved?*  ***Q5:*** *Does your country gather data to monitor uptake on telemedicine services in general and specifically in cancer care (i.e., uptake of teleconsultation services, online prescriptions)? If, yes, which kind of data is collected, and which type of indicator is available?*  ***Q6:*** *Are there trainings regarding the use of telemedicine for healthcare workforces (nurses, doctors, counsellors). If yes are specialised trainings available in cancer care/the oncology field and has the frequency increased since COVID-19?*  ***Q7:*** *Are telemedicine services (teleconsultation) in the outpatient sector publicly paid or covered in your benefits package (e.g. by sickness fund or national health service). If yes, please describe how****?*** |

**Table S1. Coding Scheme**

| **Code** | **Description** | **Examples** |
| --- | --- | --- |
| **Pre-COVID-19** | Context and implementation of telemedicine before the COVID-19 pandemic. | Existing policies, infrastructure, telemedicine usage rates, and initial challenges. |
| **COVID-19 Acceleration** | Changes and developments in telemedicine during the COVID-19 pandemic. | New policies, rapid adoption, increased funding, and accelerated implementation efforts. |
| **Perceived Benefits** | Positive impacts and advantages of telemedicine as identified by participants. | Improved access to care, continuity of care for immunocompromised patients, cost savings. |
| **Barriers and Challenges** | Obstacles and difficulties in implementing telemedicine. | Technological issues, regulatory barriers, data privacy concerns, digital divide. |
| **Facilitating Factors and Solutions** | Factors that support telemedicine implementation and proposed solutions to challenges. | Stakeholder engagement, training programs, policy recommendations, infrastructure investments. |

**Alt text Table S1:** This table outlines the coding scheme used in the study, categorising themes such as pre-COVID-19 context, COVID-19 acceleration, perceived benefits, barriers, and facilitating factors. Each code includes a description and examples, such as policies, infrastructure, challenges, stakeholder engagement, and proposed solutions.

**Case countries eHealth indicators in comparison**

Digitisation of the healthcare system can be benchmarked in various ways. Denmark is typically considered a frontrunner, a pattern that persists in benchmarking surveys. In a 2018 survey on the deployment of eHealth among general practitioners (See Table 1.), where the scale ranged from 0 (‘No awareness’) to 4 (‘Full adoption’), Denmark led, followed by Italy, Belgium, and Austria, with Poland ranking the lowest. The survey was based on OECDs definition of four categories of ICT in healthcare: Electronic Health Records, Health Information Exchange, Telehealth and Personal Health Records.

**Table S2. eHealth dimensions in the selected case countries (2018)**


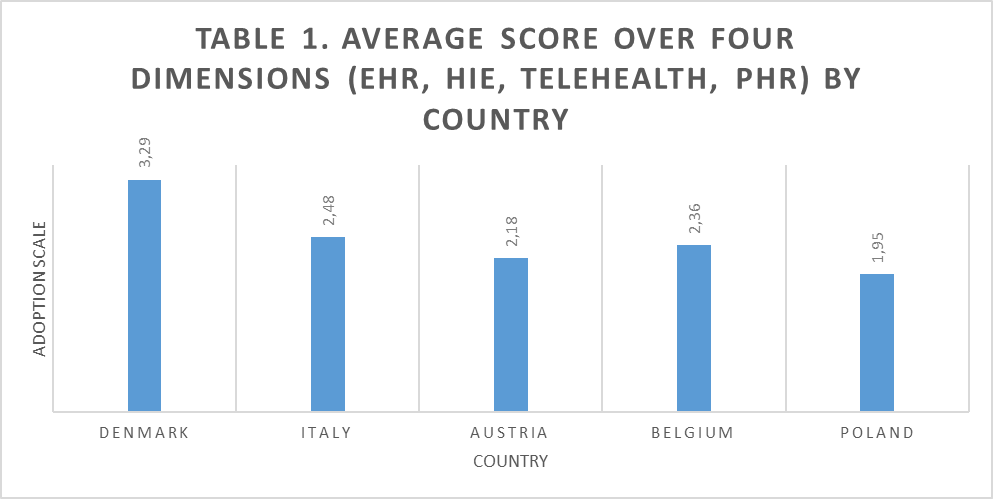


**Alt text S3**: This bar chart presents a comparison of eHealth dimensions across five case countries (Denmark, Italy, Belgium, Austria, Poland) in 2018, based on general practitioners' deployment of ICT in healthcare. Denmark leads in all categories (Electronic Health Records, Health Information Exchange, Telehealth, Personal Health Records), while Poland ranks lowest.

Source: [Benchmarking Deployment of eHealth among General Practitioners (2018)](https://op.europa.eu/en/publication-detail/-/publication/d1286ce7-5c05-11e9-9c52-01aa75ed71a1/language-en)

A similar trend emerges in a benchmarking survey on eHealth in acute hospitals (see table 2). Notably, Austria outperforms Italy and Belgium in the hospital sector, while Poland remains the lowest-ranking country. An intriguing point is the disparity between ‘Deployment’ and ‘Availability & Use’, indicating that despite having an eHealth infrastructure, there is a need to focus on the deep implementation of eHealth solutions. A crucial consideration is the data's timeframe—2018 and 2012—which may limit direct comparisons.

**Table S3. eHealth indicators in the five countries**
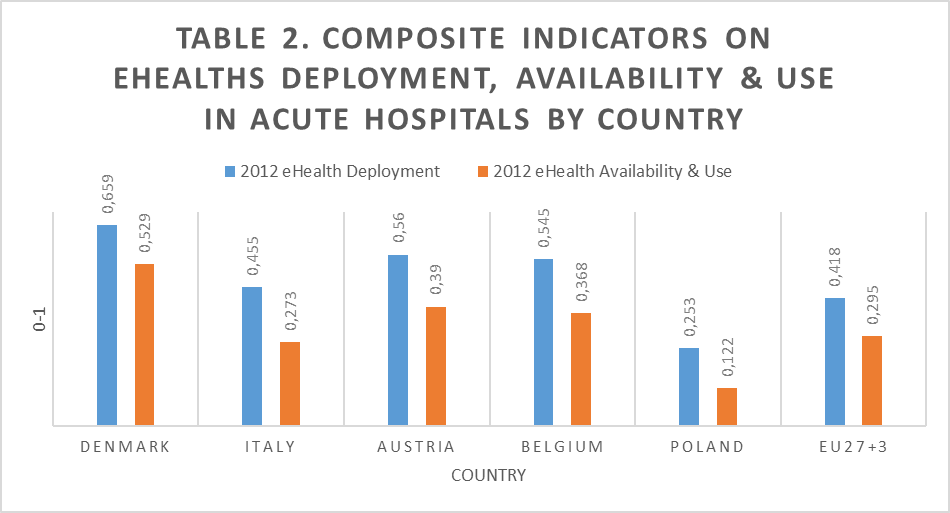


**Alt text S3:** This bar chart compares eHealth indicators in five countries (Austria, Italy, Belgium, Denmark, Poland) for hospital settings, highlighting disparities between deployment and availability/use of eHealth services. Austria outperforms Italy and Belgium in hospital sector eHealth, while Poland consistently ranks lowest. Data reflects trends from 2012–2013

Source: [European Hospital Survey - Benchmarking Deployment of eHealth services (2012-2013](https://digital-strategy.ec.europa.eu/en/library/european-hospital-survey-benchmarking-deployment-ehealth-services-2012-2013)).
